# An Enzyme-Generated Fragment of Tau Measured in Serum Shows an Inverse Correlation to Cognitive Function

**DOI:** 10.1371/journal.pone.0064990

**Published:** 2013-05-22

**Authors:** Kim Henriksen, Yaguo Wang, Mette G. Sørensen, Natasha Barascuk, Joyce Suhy, Jan T. Pedersen, Kevin L. Duffin, Robert A. Dean, Monika Pajak, Claus Christiansen, Qinlong Zheng, Morten A. Karsdal

**Affiliations:** 1 Nordic Bioscience Biomarkers and Research, Herlev, Denmark; 2 Nordic Bioscience, Beijing, China; 3 CCBR-Synarc, San Francisco, California, United States of America; 4 Neurodegeneration, H. Lundbeck A/S, Copenhagen, Denmark; 5 Translational Sciences Department, Eli Lilly and Company, Indianapolis, Indiana, United States of America; McGill University/Douglas Mental Health Univ. Institute, Canada

## Abstract

**Objective:**

Alzheimer's disease (AD) is a devastating neurological disease characterized by pathological proteolytic cleavage of tau protein, which appears to initiate death of the neurons. The objective of this study was to investigate whether a proteolytic fragment of the tau protein could serve as blood-based biomarker of cognitive function in AD.

**Methods:**

We developed a highly sensitive ELISA assay specifically detecting an A Disintegrin and Metalloproteinase 10 (ADAM10)-generated fragment of tau (Tau-A). We characterized the assay in detail with to respect specificity and reactivity in healthy human serum. We used samples from the Tg4510 tau transgenic mice, which over-express the tau mutant P301L and exhibit a tauopathy with similarities to that observed in AD. We used serum samples from 21 well-characterized Alzheimer's patients, and we correlated the Tau-A levels to cognitive function.

**Results:**

The Tau-A ELISA specifically detected the cleavage sequence at the N-terminus of a fragment of tau generated by ADAM10 with no cross-reactivity to intact tau or brain extracts. In brain extracts from Tg4510 mice compared to wt controls we found 10-fold higher levels of Tau-A (p<0.001), which indicates a pathological relevance of this marker. In serum from healthy individuals we found robust and reproducible levels of Tau-A, indicating that the analyte is present in serum. In serum from AD patients an inverse correlation (R^2^ = 0.46, p<0.001) between the cognitive assessment score (Mattis Dementia Rating Scale (MDRS)) and Tau-A levels was observed.

**Conclusion:**

Based on the hypothesis that tau is cleaved proteolytically and then released into the blood, we here provide evidence for the presence of an ADAM10-generated tau fragment (Tau-A) in serum. In addition, the levels of Tau-A showed an inverse correlation to cognitive function, which could indicate that this marker is a serum marker with pathological relevance for AD.

## Introduction

Alzheimer's disease (AD) is a devastating neurological disease, which with the ever-increasing age of the population is expected to explode in numbers. AD is characterized by global cognitive decline including language breakdown. At present, treatment is limited to alleviation of the symptoms and disease modifying approaches have so far failed [1]–[3]. A contributing factor to the lack of success within drug development is the absence of blood-based biomarkers, which indicate disease progression and thereby can help the selection of patients for clinical trials [4].

Hence, methods allowing monitoring of neurodegeneration in AD, i.e. before onset of cognitive loss, are intensely sought, as these are essential to design clinical trials assessing the potential of drugs to prevent progression of AD [4].

Cerebrospinal fluid (CSF) biomarkers have provided diagnostic value for AD; however, their application is limited owing to the invasiveness of lumbar puncture [4].

Potential candidate biomarkers are protein fragments which reflect specific cleavage sites in proteins, and, due to their smaller size, may pass the Blood-Brain-Barrier (BBB) and thereby be detected in serum [5]. Importantly, these smaller protein fragments may yield more information than their intact counterparts because they have been degraded by specific enzymes, which may be an important feature of AD [6], [7].

In AD, the pathological processing of the protein Tau by proteases is of great interest [8], as this appears to be a key correlate of neuronal cell death [6]. Proteolytic cleavage of tau is mediated by several different proteases, such as caspases and calpains [8]. However, several other proteases also appear to play a role in neuronal degeneration even though they mainly have been associated with secretase functions [8].

We hypothesized that a link between plaques and NFTs involves a process in which the intracellular tau protein is exposed to extracellular or even circulating secretases, such as ADAM10, i.e. during neuronal apoptosis [6]. Secondly, we hypothesized that this secretase-mediated cleavage of tau would lead to the generation of fragments which could be used as biomarkers of AD.

We thus aimed to develop a useful serum assay monitoring a tau degradation fragment generated by ADAM10, a putative α-secretase [8] and assessed the pathological relevance of this assay by its ability to detect tau degradation fragments in rodent samples, as well as human serum samples collected from both healthy individuals and from Alzheimer's patients.

## Materials and Methods

### In Vitro cleavage for mass spectrometry

Protease cleavage was performed by mixing 100 µg tau and 1 µg of enzyme (ADAM10) MMP buffer (100 mM Tris-HCl, 100 mM NaCl, 10 mM CaCl_2_, 2 mM Zn acetate, pH 8.0) and incubating for 7 days. Finally the cleavage was verified by visualization using the SilverXpress*®* Silver Staining Kit (cat. no. LC6100, Invitrogen, Carlsbad, Ca, USA) according to the manufacturer's instructions.

### Peptide identification

Peptide fragments in the *in vitro* cleaved samples were identified using matrix-assisted laser desorption time of flight mass spectrometry (MALDI-TOF MS) and liquid chromatography coupled to electro spray ionization (ESI) tandem mass spectrometry (LC-MS/MS). MALDI-TOF samples were purified using C18 zip-tips (cat.no.ZTC18SO24, Millipore, Billerica, MA, USA) according to specifications and 0.1 µg of material was eluted onto a MTP 384 ground steel target plate (Bruker-Daltonics, Bremen, Germany). MALDI tandem mass spectra were recorded on a Bruker ultraflex MALDI-TOF/TOF mass spectrometer (Bruker-Daltonics, Bremen, Germany) in positive ion reflector mode. Mass spectra were externally calibrated in the *m*/*z* range of 800−4000 using peptides generated by tryptic digestion of bovine β-lactoglobulin. The m/z software “Flexanalysis” (Bruker-Daltonics, Bremen, Germany) was used to analyze spectra. LCMS samples were ultra-filtrated to remove proteins above 10 kDa, the pH was adjusted to 2.0 using formic acid, and a 4 µL sample was analyzed by LC-MS/MS. LC was performed on a nanoACQUITY UPLC BEH C18 column (Waters, Milford, MA, USA) using a formic acid/acetonitrile gradient. MS and MS/MS were performed on a Synapt High Definition Mass Spectrometry quadruple time of flight MS (QUAD-TOF; Waters, Milford, MA, USA), with acquisition range of 350-1600 m/z in MS and 50-2000 m/z, in MS/MS. The software “ProteinLynx Global SERVER (PLGS)” (Waters, Milford, MA, USA) was used to analyze spectra and generate peak lists. To identify peptides, MS and MS/MS data were searched against a tau (FASTA) protein database using the Mascot 2.2 (Matrix Science, Boston, MA, USA) software with either the MALDI-TOF/TOF or ESI-QUAD-TOF settings.

### Selection of peptide for immunizations

The first six amino acids of each free end of the sequences identified by MS were regarded as neo-epitopes generated by the protease in question. All obtained protease-generated sequences were analyzed for homology and distance to other cleavage sites and then blasted for homology using the NPS@:network protein sequence analysis, and from these analyses three unique cleavage fragments were selected.

### Reagents and peptides

All reagents were standard high-quality chemicals from companies such as Merck and Sigma Aldrich. The synthetic peptides used for monoclonal antibody production and validation were: Immunogenic peptide:

TPRGAAPPGQ-GGC-KLH (Keyhole-Limpet-Hemocyanin)

Selection/screening peptide

TPRGAAPPGQ

De-selection peptide:

**A**TPRGAAPPGQ

Deselection peptides elongated with one amino acid in the N-terminus were purchased from the Chinese Peptide Company, Beijing, China. Peptide conjugation reagents were produced by Pierce (Thermofisher, Denmark).

### Development of the antibody and ELISA

The methods used for monoclonal antibody development were as previously described [9], [10].

### ELISA methodology

In preliminary experiments, we optimized the reagents, their concentrations and the incubation periods by performing several checkerboard analyses (data not shown).

The final Tau-A ELISA was developed as follows: A 96-well ELISA plate pre-coated with streptavidin was further coated with 6 ng/ml of the synthetic peptide TPRGAAPPGQ-Biotin dissolved in Tris-BT buffer at 20°C for 30 min by constant shaking at 300 rpm. The plate was washed five times in washing buffer and 20 µl of sample was added, followed by 100 µl of peroxidase conjugated anti-human mAb-Tau-A solution (50 ng/ml). The plate was incubated for 1 h at 20°C in assay buffer (150 mM Trizma, 1% BSA, 0.05% Tween-20, 0.36% Bronidox L5) during which time it was shaken at 300 rpm.

The plate was again washed five times followed by the addition of 100 µl tetramethylbenzinidine (TMB) (Kem-En-Tec cat.438OH). The plate was incubated for 15 min in darkness and shaken at 300 rpm. In order to cease the reaction, 100 µl of stopping solution (95–97% H_2_SO_4_, Merck Cat. No.: 1.00731) was added and the plate was analysed in the ELISA reader at 450 nm with 650 nm as the reference.

### Buffers used for the ELISAs

Buffer used for dissolving the coating peptide was composed of the following: (50 mM PBS, 137 mM, 1% BSA, 0.05% Tween-20, 0.9% EDTA, 0.36% Bronidox L5, 10% sorbitol, pH = 7), and reaction stopping buffer composed of 0.1% H_2_SO_4_

ELISA-plates used for the assay development were Streptavidin-coated from Roche cat.: 11940279. All ELISA plates were analysed with the ELISA reader from Molecular Devices, SpectraMax M, (CA, USA).

### Standards

A standard curve was performed by serial dilution of the synthetic selection peptide. Synthetic standard concentrations for Tau-A were 0, 0.59, 1.17, 2.34, 4.69, 9.38, 18.75, 37.5, 75, 150, and 300 ng/ml.

### Samples for testing native reactivity of the antibodies

For assay development and validation, serum and plasma from 15 healthy adult volunteers aged 23–45 years of both genders were used. We also tested serum samples from mice and rats to determine the level of interspecies cross reactivity.

### Animal samples

Tissues including brain, liver, muscle, colon, kidney, lung, skin and pancreas isolated from 5 six-month-old Sprague Dawley rats and 5 brains from each of either the wildtype or Tg4510 mice were flash-frozen in liquid nitrogen and pulverized using a Bessman pulverizer. The “powder” was transferred to a vial and weighed. Extraction buffer (50 mM Tris-HCl, 50 mM HEPES, 1 mM EDTA, 0.5% sodium deoxycholate, 15% glycerol, protease inhibitor cocktail (Roche cat#05056489001), pH 8.3) was added at 1 mL buffer/250 mg tissue. The lysate was cleared by sonication. After sonication the debris was spun at 4°C/5 min/10000 rpm and the supernatants were collected and stored at −80°C until further use.

Protein concentrations were determined using the DC Protein Assay (Biorad).

### In Vitro cleavage of tissues

Protease cleavage was performed by mixing 100 µg of tissue extract and 1 µg of ADAM10 in MMP buffer (100 mM Tris-HCl, 100 mM NaCl, 10 mM CaCl_2_, 2 mM Zn acetate, pH 8,0) and incubating for 7 days. Finally, the cleavage was verified by western blotting and ELISA analysis.

### Western Blotting

20 µg of each rat tissue extract and 100 µg of each mouse tissue extract was loaded onto an SDS-PAGE gel, and the gel was run, followed by transfer of the proteins to nitrocellulose membranes. Ponceau Red staining was then used to verify equal protein loading on the membranes. The levels of Tau-A fragments and total tau protein were detected by incubation with the primary antibodies diluted to 100 ng/mL in TBS-T containing skim milk powder [11]. A secondary antibody recognizing mouse IgG conjugated to horse-radish peroxidase was then added, and finally the blot was visualized using enhanced chemiluminescence as previously described [11].

### Human samples

Aliquots of serum collected at baseline were obtained from Alzheimer's disease (AD) patients (n = 21) that participated in a clinical trial entitled “*A Prospective, Randomized Start, Multicenter, Double-Blinded, Concurrently Placebo-Controlled Study to Evaluate the Effect of Flow-Regulated Ventriculoperitoneal Shunting on Progression of Alzheimer's disease: An Investigation of the Safety and Effectiveness of the COGNIshunt™ CNS Fluid Shunt System*” sponsored by Eunoe, Inc. (Pleasanton, CA). The trial is registered on clinicaltrials.gov with the identified: NCT00056628. Characteristics: Age at onset 70(SD+/−7), Females/Males (16/6), baseline MDRS score 112(SD+/−12), Intact tau measured in baseline CSF samples (collected at shunt implantation) was 849 pg/mL (SD+/−1009 pg/mL). Further details of the study were previously reported by Silverberg et al [12]. Subject inclusion required a clinical diagnosis of probable AD based on National Institute of Neurological Disorders and Stroke–Alzheimer's Disease and Related Disorders Association (NINCDS-ADRDA) criteria [13] and a Mini-Mental State Examination (MMSE) score between 15 and 24, inclusive. Blood collected at baseline by venipuncture was allowed to clot and serum was prepared by centrifugation. Serum was stored frozen at −70°C until analyzed. Results derived from analysis of serum was compared to baseline Mattis Dementia Rating Scale (MDRS) scores obtain before initiation of study intervention [14], [15].

### Ethics

Animal samples were from a previously published study [16].

Study subjects and either a family member or a Durable Power of Attorney (if one existed) gave written informed consent including a special addendum permitting the use of stored specimens for AD research [12]. According to Danish legislation no ethical approval is needed to perform the analysis of serum samples collected in a prior study, and a waiver describing this is on file at Nordic Bioscience.

### Statistical analysis

For assay validation, optical density was fitted against analyte concentration applying a four-parameter logistic regression to the calibration curve. Average, standard deviations, percentage coefficient of variation (%CV), and differences from theoretical values were calculated for all standards and samples. Quantitative data were analysed using GraphPad Prism 5 (GraphPad Software, San Diego, CA, USA). Significant differences between means were determined using Student's two-tailed unpaired t-test. Normality of the data was ensured using a Shapiro-Wilkes Test. Data were expressed as mean (or geometric means) ± standard error of the mean and differences were considered significant at a p level of 0.05 or lower.

## Results

### Characterization of the Tau-A ELISA assay

**Figure 1:**
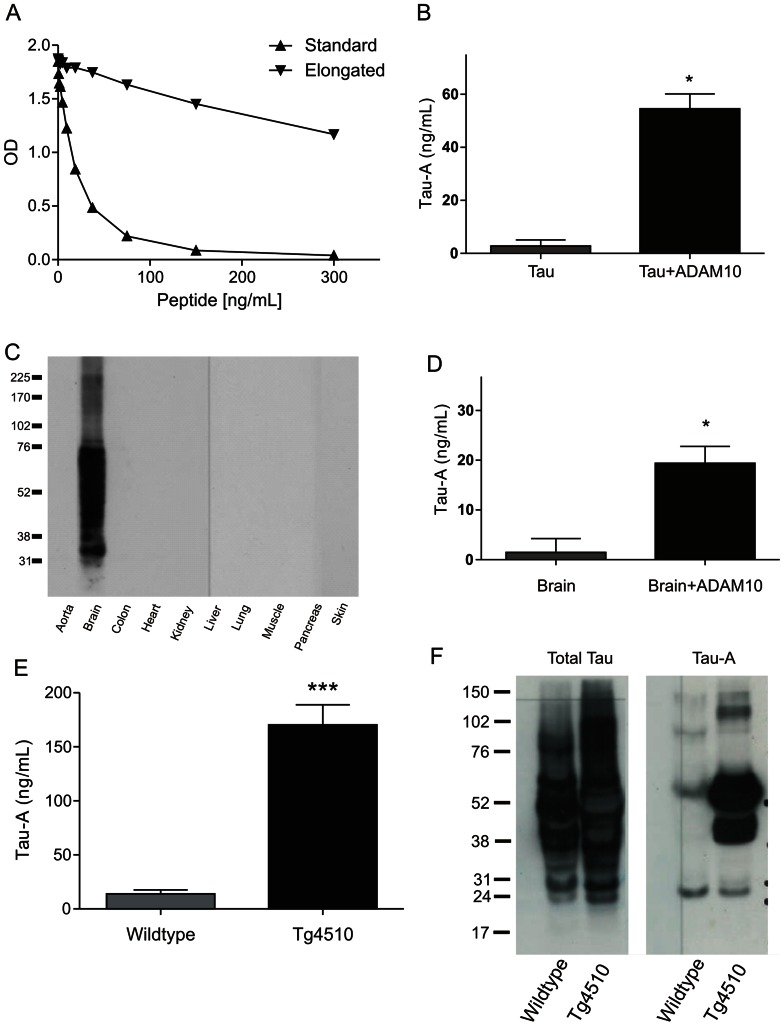
Characteristics and biological validation of the Tau-A assay. A) Standard curves with either the selection peptide or the elongated peptide at concentrations of 0, 0.59, 1.17, 2.34, 4.69, 9.38, 18.75, 37.5, 75, 150, and 300 ng/ml. B) Measurement of Tau-A fragments in *in vitro* digests of tau. C) Western blots of extracted tissues, D) ELISA measurement of brain extracts in the presence or absence of ADAM10. E) Tau-A levels in extracted brains from either control or Tg4510 mice (a model of Alzheimer's disease) measured using the ELISA. F) Western blots comparing brain extracts from wild type and Tg4510 mice. Left: a western blot conducted with an antibody recognizing intact tau (MAB3420 Chemicon). Right: Western blot conducted with our in-house antibody (NB191).

An antibody recognizing the ADAM10 generated cleavage sequence of tau (TPRGAAPPGQ) was raised, and used for development of an ELISA (Tau-A). As seen in figure 1, the assay is specific for the cleavage site, as an extension of the sequence by one amino acid led to loss of reactivity (Figure 1A). Further validation of specificity using ADAM10 degraded recombinant tau or brain extracts confirmed the specificity towards cleaved tau (Figure 1B–C). In addition, tissue profiling confirmed that tau primarily originates from the brain. The lower limit of detection (LLOD) was determined to be 2.9 ng/mL and the upper limit of detection (ULOD) was 226.3 ng/mL. The assay was technically robust and was able to detect Tau-A levels in human serum and plasma, as well as mouse and rat serum within dilution ranges of 1+2 to 1+6 (Table 1). In addition, a linear spiking recovery was obtained within the above described dilution ranges (data not shown). The intra-assay coefficient of variation was 5.8%, while the inter-assay CV% was 12.6%. No loss of reactivity was observed following 5 consecutive freeze-thaw cycles.

**Table 1:** Dilution recovery measured in human samples and calculated in % of lowest dilution possible.

Tau-A ng/mL	HS1 45.2	HS2 33.7	HS3 15.8	Average	HP1 48.0	HP2 30.3	HP3 36.9	Average
Diluted 1+2	100	100	100		-	-	-	
Dilution 1+3	90	94	118		100	100	100	
Dilution 1+4	90	96	114		103	112	98	
Dilution 1+5	80	83	108		107	116	111	
Dilution 1+6	81	78	107		111	102	109	
Dilution 1+7	-	-	-		104	95	97	
Mean	85	88	112	95	107	106	104	105

Tau-A ng/mL	RS1 135.8	RS2 48.6	RS3 83.3	Average	MS1 24.4	MS2 14.1	MS3 13.6	Average
Dilution 1+2	100	100	100		100	100	100	
Dilution 1+3	106	113	98		105	84	111	
Dilution 1+4	80	107	94		93	87	111	
Dilution 1+5	83	105	94		108	103	137	
Dilution 1+6	80	98	101		-	-	-	
Mean	87	106	97	97	102	91	111	101

Values in bold represent data points outside the accepted variation and are excluded from the calculations as they are measured too close to the lower limit of detection (LLOD) value (2.9 ng/mL). HS = Human Serum, HP = Human Plasma. RS = Rat Serum, MS = Mouse Serum.

### Biological validation of the Tau-A ELISA

Analysis using the ELISA revealed that brains from the animal model of tauopathy, the Tg4510 mice, had 10-fold higher levels of Tau-A than their corresponding wild type (wt) controls (Figure 1E). Western blotting also showed that Tg4510 mice had very high levels of Tau-A, while the control mice had very little (Figure 1F), even if equal amounts of protein were loaded (data not shown). Re-probing the blot with an antibody against total tau showed almost equal levels of total tau, although the intensity of the high molecular weight bands was greater in the Tg4510, as expected from the model [16].

### Tau-A levels correlate with MDRS score

**Figure 2:**
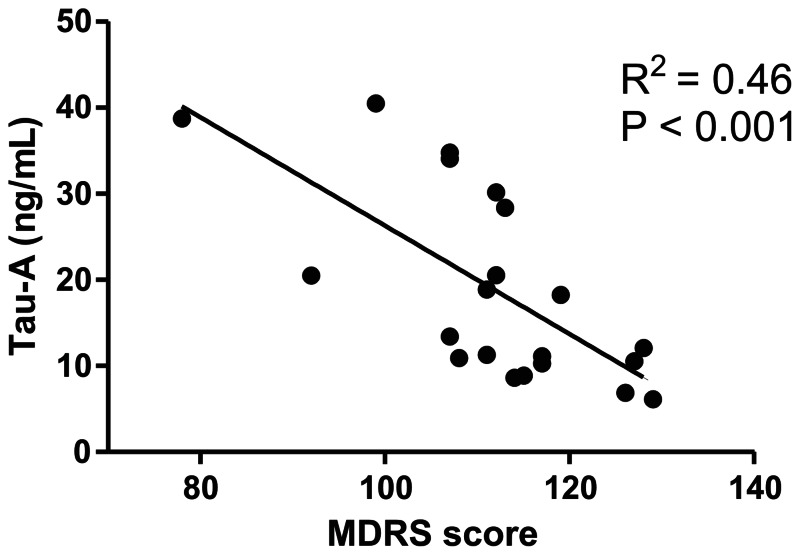
Pathology related changes in Tau-A levels. Inverse correlation between Tau-A and the Mattis Dementia Rating Scale (MDRS).

To investigate whether a relationship between the marker and AD disease stage, we correlated the tau levels in the AD patients to scores obtained using the Mattis Dementia Rating Scale [17], and highly interestingly we found a significant (p = 0.003) and inverse relationship between MDRS and Tau-A (Figure 2). No correlations to other parameters, such as intact tau in CSF or age could be observed in this small cohort (data not shown).

## Discussion

Potential serum and/or plasma-based markers for AD have been investigated extensively, yet a single biomarker with a correlation to cognitive function remains to be identified [4].

In this study, we developed an ELISA assay detecting fragments of tau generated by ADAM10 with the exclusive aim of analyzing pathological tau processing in serum. Firstly, we confirmed that the antibody was highly specific towards an ADAM10 generated fragment of tau, and that this fragment was detectable in serum. In terms of absolute amount of the analyte in blood, this is unknown, although it likely is low; however, a determination of these will require further study, as the assay employs a synthetic standard.

Importantly, this marker showed an inverse correlation with MDRS, indicating that it is related to loss of cognitive function. A biochemical marker monitoring proteolytic processing of tau in serum, is of interest as this is a key process in the development of cognitive deficits [6], [8]. It is also the first serum tau biomarker which correlates to cognitive function, thereby showing promise for the application of this marker in future studies. While other studies have indicated that Aβ levels in plasma are correlated to cognitive function, albeit in elderly without dementia [18], this is still the first study measuring tau processing

While neo-epitopes are nothing new in AD, as measurements of Aβ42 and phosphorylated tau have been reported as neo-epitopes formed as a consequence of disease [4], selective screening of serum for *in vitro* generated tau fragments had not previously been published.

The combination of ADAM10 and tau selected for this work was based on the novel hypothesis that during progression of AD, tau will be exposed to secretase-mediated cleavage either directly in the brain or as fragments generated by other brain proteases, which then become secondarily processed as they enter the circulation; however, this requires further studies. We were able to detect a specific ADAM10-generated tau peptide fragment in serum, as well as highly elevated levels in the brains of the Tg4510 mice, a model of tauopathies known to demonstrate tau aggregation [16]. These observations suggest ADAM10 processing of tau is a relevant process in neuronal death during tauopathies, such as AD, although the exact mechanism of action still remains to be identified.

Using mass spectrometry we identified the sequence ^470^TPRGAAPPGQ which is derived from the brain isoform of tau and highly specific for tau. Interestingly, this neo-epitope resides in a part of the tau sequence which is speculated to be removed by processing during the progression of AD, and thus may be of pathological relevance [19].

To ensure that the identified tau fragment was specific to neurons, and not found in other tissues, we performed a series of extractions, and we found that total tau is primarily expressed in brain, and that the addition of ADAM10 led to significant cleavage of intact tau. Tau-A was not detected in any other intact or digested tissues, supporting the tissue- specificity of the fragment. In brain extracts of the tauopathy model, the Tg4510 mice, we found 10-fold elevated levels of Tau-A compared with healthy controls, indicating pathological relevance.

Importantly, we found that Tau-A levels correlated inversely with MDRS, a finding which supports that Tau-A to loss of cognitive ability [17], [20], albeit in a fairly small group of patients. Whether this correlation directly reflects loss of neurons requires further studies using more direct measurements of neuronal loss, which could be a key aspect in determining whether this marker can be applied to identify earlier progressors, such a those with prodromal AD. On the other hand we found no correlation to total tau in CSF or to age (data not shown), but these findings may be explained by the small sample size. While tau pathology is clearly related to AD, it is well-established that pathological tau processing occurs in several different forms of dementia, and hence further studies in other dementias as well as cognitively normal elderly are needed to further characterize the potential of this marker.

In summary, we have developed the first serum-based assay detecting pathological fragments of tau, and importantly this fragment was directly and inversely related to cognitive function. We speculate that the assay could be a useful and practical tool for the diagnosis of neuronal loss, and could monitor the efficacy of treatment and progression of AD.
